# Trimetazidine Protects against Smoking-Induced Left Ventricular Remodeling via Attenuating Oxidative Stress, Apoptosis, and Inflammation

**DOI:** 10.1371/journal.pone.0040424

**Published:** 2012-07-06

**Authors:** Xiang Zhou, Chao Li, Weiting Xu, Jianchang Chen

**Affiliations:** 1 Department of Cardiology, The Second Affiliated Hospital of Soochow University, Suzhou, China; 2 Department of Medicine, The University of Hong Kong, Pokfulam, Hong Kong, China; National Institutes of Health, United States of America

## Abstract

Trimetazidine, a piperazine derivative used as an anti-anginal agent, improves myocardial glucose utilization through inhibition of fatty acid metabolism. The present study was designed to investigate whether trimetazidine has the protective effects against smoking-induced left ventricular remodeling in rats. In this study, Wistar rats were randomly divided into 3 groups: smoking group (exposed to cigarette smoke), trimetazidine group (exposed to cigarette smoke and treated with trimetazidine), and control group. The echocardiographic and morphometric data indicated that trimetazidine has protective effects against smoking-induced left ventricular remodeling. Oxidative stress was evaluated by detecting malondialdehyde, superoxide dismutase, and glutathione peroxidase in the supernatant of left ventricular tissue. Cardiomyocyte apoptotic rate was determined by flow cytometry with Annexin V/PI staining. Gene expression and serum levels of inflammatory markers, including interleukin-1β, interleukin-6, and tumor necrosis factor-α, were deteced by quantitative real-time PCR and enzyme-linked immunosorbent assay. Our results suggested that trimetazidine could significantly reduce smoking-induced oxidative stress, apoptosis, and inflammation. In conclusion, our study demonstrates that trimetazidine protects against smoking-induced left ventricular remodeling via attenuating oxidative stress, apoptosis, and inflammation.

## Introduction

Smoking, which is a serious public health concern, contributes significantly to cardiovascular morbidity and mortality [1]–[4]. Cigarette smoke contains more than 4,000 chemical substances, including polycyclic aromatic hydrocarbons and oxidative gases, most of which exert a cardiotoxic effect. Nicotine is the addictive component and most harmful ingredient contained within cigarettes. In a previous study, we concluded that nicotine promotes cardiomyocyte apoptosis by inducing oxidative stress and disrupting apoptosis-related gene expression [5].

Ventricular remodeling, defined as changes in size, shape and function of the heart in response to cardiac injury or increased load, is associated with the development and progression of heart failure. The process of ventricular remodeling is largely influenced by haemodynamic load, neurohumoral activation and additional factors such as endothelin, cytokines, nitric oxide production and oxidative stress [6]. In the past few years, several studies have demonstrated that exposure to cigarette smoke can result in cardiac remodeling and impaired ventricular function [7]–[9].

Trimetazidine, a piperazine derivative used as an anti-anginal agent, selectively inhibits long-chain 3-ketoacyl coenzyme A thiolase (the last enzyme involved in β-oxidation) activity. Previous studies have demonstrated that trimetazidine can improve left ventricular function in patients with heart failure [10]–[12]. Trimetazidine may affect myocardial substrate use by inhibiting oxidative phosphorylation and shifting energy production from free fatty acids to glucose oxidation [13]. It may also contribute to the preservation of intracellular levels of phosphocreatine and ATP [14], reduce free radical-induced injury [15], inhibit cell apoptosis [16] and improve endothelial function [17]. The present study was designed to investigate whether trimetazidine has the protective effects against smoking-induced left ventricular remodeling in rats.

## Materials and Methods

### Ethics statement

This study was carried out in accordance with the National Institute of Health's Guide for the Care and Use of Laboratory Animals and was approved by the Animal Ethics Committee of Soochow University (Protocol number SD2011288).

### Groups and treatment

Male Wistar rats weighing 200–250g were housed two per plastic cage with wood chips for bedding in an animal room with an alternating 12 h light/dark cycle at 22±2°C and 55±10% relative humidity. The rats were randomly divided into 3 groups: smoking group (n = 10), exposed to cigarette smoke at the rate of 40 cigarettes/day for 4 months; trimetazidine group (n = 10), exposed to cigarette smoke (40 cigarettes/day) and meanwhile treated with trimetazidine (10 mg/kg/day, Les Laboratoires Servier) for 4 months; control group (n = 10), neither exposed to cigarette smoke nor treated with trimetazidine. Drugs were prepared from the tablets, suspended in 0.9% NaCl and applied by stomach tube in a volume of 2.0 ml/kg/day.

The rats were exposed to cigarette smoke in a chamber (dimensions 95×80×65cm) connected to a smoking device according to the method proposed by Wang, et al. [18]. The smoke was drawn out of filtered commercial cigarettes (composition per unit: 1.1mg of nicotine; 12mg of tar; and 13mg of carbon monoxide) with a vacuum pump and was exhausted into the smoking chamber. During the first week, the number of cigarettes was gradually increased from 5 to 10 cigarettes over a 30-min period, twice in the afternoon. After that, 10 cigarettes were used in each smoking trial, repeated 4 times/day, twice in the morning and twice in the afternoon.

**Table 1:** Echocardiographic study.

	Control group	Smoking group	Trimetazidine group
Heart rate (bpm)	273±16	284±25	291±22
LVEDD (mm)	7.50±0.22	8.13±0.30*	7.81±0.24**
LVESD (mm)	4.16±0.20	4.71±0.31*	4.43±0.25**
LVPWT (mm)	1.25±0.11	1.36±0.16	1.32±0.18
FS	0.45±0.02	0.40±0.03*	0.43±0.02**
EF	0.88±0.03	0.82±0.03*	0.85±0.02**
E/A	1.53±0.16	1.32±0.21	1.46±0.19
Ea/Aa	1.42±0.23	1.27±0.25	1.35±0.31

LVEDD: left ventricular end-diastolic dimension; LVESD: left ventricular end-systolic dimension; LVPWT: left ventricular posterior wall thickness; FS: fractional shortening; EF: ejection fraction; E: peak velocity of early ventricular filling; A: peak velocity of transmitral flow during atrial contraction. Ea: early diastolic mitral annular velocity; Aa: late diastolic mitral annular velocity. Data are expressed as mean ± SD. *P<0.05, vs. control group; **P<0.05, vs. smoking group.

**Table 2:** Morphological data.

	Control group	Smoking group	Trimetazidine group
BW (g)	457±31	438±23	446±27
LVW/BW (mg/g)	2.16±0.14	2.47±0.18*	2.32±0.17**
CSA (μm^2^)	245±8	263±11*	254±7**
ICVF (%)	3.36±0.26	4.03±0.29*	3.72±0.28**

BW: body weight; LVW: left ventricular weight; CSA: cross-sectional area; ICVF: interstitial collagen volume fraction. Data are expressed as mean ± SD. *P<0.05, vs. control group; **P<0.05, vs. smoking group.

### Echocardiographic study

After 4 months, transthoracic echocardiography was carried out to evaluate cardiac structure and function in these 3 groups. All echocardiograms were performed according to the American Society of Echocardiography guidelines [19]. Echocardiograms were conducted by the same experienced sonographer using an Acuson Sequoia C256 Echocardiography System (Acuson Corp., Mountain View, CA) and a 15.0 MHz transducer. Rats were lightly anesthetized by intramuscular injection with a mixture of ketamine (50mg/kg) and xylazine (1mg/kg). The transducer was placed on the left thorax, and M-mode and 2-dimensional echocardiography images were obtained in the parasternal long-axis views. The cardiac structures were measured in 3 to 5 consecutive cardiac cycles as follows: left ventricular end-diastolic dimension (LVEDD) and left ventricular posterior wall thickness (LVPWT) were measured at maximal diastolic dimension, and left ventricular end-systolic dimension (LVESD) was taken at the maximal anterior motion of the posterior wall. Left ventricular systolic function was assessed by calculating fractional shortening [FS  =  (LVEDD-LVESD)/LVEDD], and ejection fraction [EF  =  (LVEDD^3^-LVESD^3^)/LVEDD^3^]. The transmitral diastolic flow (E and A waves) was obtained with the transducer at the apical 4-chamber view. The peak velocities in the rapid ventricular filling phase (E wave) and during atrial contraction (A wave) were measured in 5 consecutive cardiac cycles and were averaged. Tissue Doppler imaging recordings were obtained from the lateral mitral valve annulus. Early diastolic (Ea) and late diastolic (Aa) mitral annular velocities were measured in 5 consecutive cardiac cycles and the results were averaged. The E/A and Ea/Aa ratios were calculated and used as indices of left ventricular diastolic function.

### Morphometric analysis

All animals were weighed, euthanized by carbon dioxide inhalation, then left ventricles (including the interventricular septum) were dissected, separated, and weighed. Left ventricular mass index (LVMI) was calculated as left ventricular mass divided by the rat's body weight. Left ventricular tissue was fixed in 10% buffered formalin and embedded in paraffin. Five-micron-thick sections were stained with hematoxylin-eosin (HE) and with the collagen-specific stain picrosirius red. Myocyte cross-sectional area (CSA) was determined for at least 100 myocytes per slide stained with HE. The measurements were performed using a Leica microscope (magnification lens ×400) attached to a video camera and connected to a personal computer equipped with image analyzer software (Image-Pro Plus 3.0, Media Cybernetics, Silver Spring, MD). CSA was measured with a digitizing pad, and the selected cells were transversely cut with the nucleus clearly identified in the centre of the myocyte. Interstitial collagen volume fraction (ICVF) was determined for the entire picrosirius red-stained cardiac section using an automated image analyzer (Image-Pro Plus 3.0, Media Cybernetics). Cardiac tissue components were identified according to the colour level: red for collagen fibres, yellow for myocytes, and white for interstitial space. The digitized profiles were sent to a computer that calculated collagen volume fraction as the sum of all connective tissue areas divided by the sum of all connective tissue and myocyte areas. On average, 35 microscopic fields were analyzed with a 40× lens. Perivascular collagen was excluded from this analysis.

**Figure 1:**
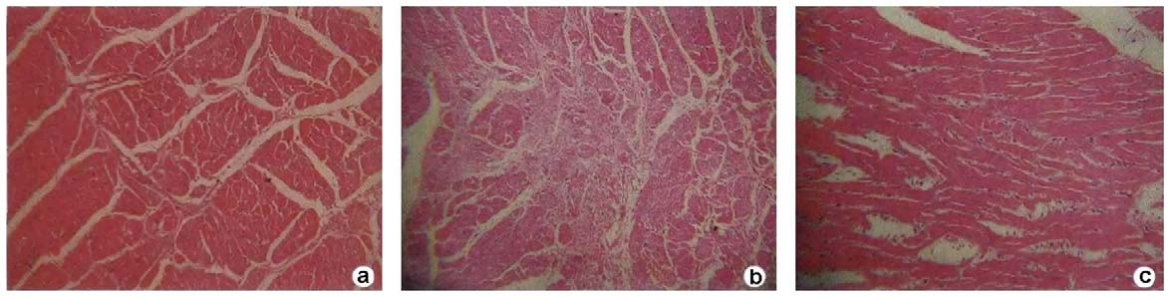
HE-stained pathological images of left ventricular tissue. **a**: Cardiomyocytes in the control group were orderly arranged, and the nuclei were lightly stained and located in the center of muscle fibers. **b**: Thickening and lengthening of myocardial fibers could be observed in the smoking group, wherein the nuclei were darkly stained, showing local tissue fibrosis and inflammatory cell infiltration. **c**: Cardiomyocyte hypertrophy, cellular degeneration and inflammatory cell infiltration were significantly improved in the trimetazidine group by contrast with those in the smoking group.

**Table 3:** Assessment of oxidative stress.

	Control group	Smoking group	Trimetazidine group
MDA (nmol/mg protein)	0.58±0.16	0.96±0.24*	0.77±0.19**
SOD (U/mg protein)	178.41±16.43	137.13±24.04*	157.72±20.51**
GSH-Px (U/mg protein)	256.63±17.20	209.34±21.32*	233.72±22.75**

MDA: malondialdehyde; SOD: superoxide dismutase; GSH-Px: glutathione peroxidase. Data are expressed as mean ± SD. *P<0.05, vs. control group; **P<0.05, vs. smoking group.

### Assessment of oxidative stress

Rat left ventricular tissue was removed and homogenized in 50 mmol/L phosphate buffer (pH 7.4) kept in an ice bath. The homogenates were centrifuged for 10 min at 8,500 g (4°C), and the pellet (cellular debris) was discarded. The supernatants were harvested and stored at –20°C until biochemical assays were performed. Oxidative stress was evaluated by detecting malondialdehyde (MDA), superoxide dismutase (SOD) and glutathione peroxidase (GSH-Px) in the supernatant according to the instructions of detection kits from Nanjing Jiancheng Bioengineering Institute (Nanjing, China). MDA level was measured with the thiobarbituric acid reaction using Ohkawa's method [20]. The principle of this method is the spectrophotometric measurement of the color generated by the reaction of thiobarbituric acid with MDA. SOD activity was assayed using the nitroblue tetrazolium method proposed by Sun, et al [21]. This method is based on the inhibition of nitroblue tetrazolium reduction by the xanthine-xanthine oxidase system as a superoxide generator. GSH-Px activity was measured according to the method of Paglia and Valentine [22]. Analysis of supernatant GSH-Px activity is based on oxidized glutathione produced upon reduction of an organic peroxide by GSH-Px, which is recycled to its reduced state by glutathione reductase.

### Quantitation of apoptosis by Annexin V/PI staining

Rat left ventricular tissue was surgically removed, minced into small pieces, and then digested for 10 min at 37°C with trypsin solution. 1×10^5^ cells were collected and washed twice with cold PBS. 5 μl of Annexin V (Pharmingen, San Diego, CA, USA) and 5 μl of PI (Sigma, St. Louis, MO, USA) were added to the cells, which were resuspended in 500 μl 1× binding buffer. The cells were gently vortexed and incubated for 15 min at room temperature in the dark, then they were analyzed by flow cytometry within 1 h. Annexin V labeled with a fluorophore could identify cells in the early stage of apoptosis, and PI, a fluorescent nucleic acid binding dye, was responsible for staining cells in the medium and late stages of apoptosis. Analysis was based on gating a subpopulation of cells by forward scatter versus side scatter. The intermediate to large mononuclear cell population was the gated region used to calculate the apoptotic rate. The apoptotic rate was calculated as the percentage of Annexin V-positive and PI-negative cells divided by the total number of cells in the gated region.

**Figure 2:**
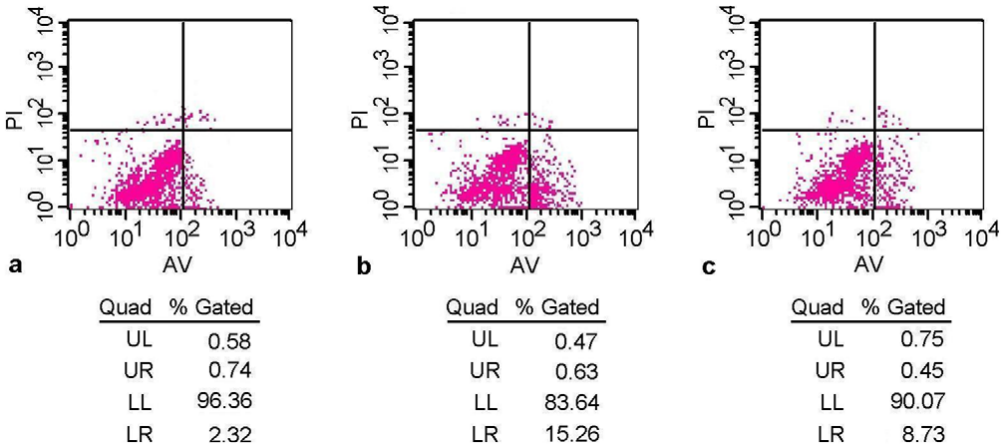
Cardiomyocyte apoptotic rate was determined by flow cytometry with Annexin V/PI staining. The apoptotic rate in the smoking group (**b**) was significantly higher than in the control group (**a**), while this rate in the trimetazidine group (**c**) was significantly lower than in the smoking group.

**Figure 3:**
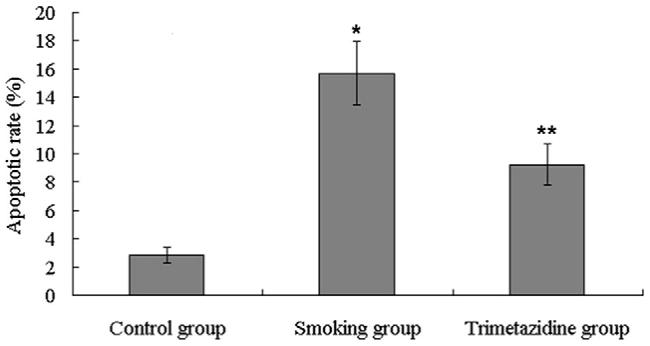
The apoptotic rate of cardiomyocytes in the smoking group increased significantly compared with that in the control group, while the apoptotic rate in the trimetazidine group declined remarkably in comparison with that in the smoking group. *P<0.05, vs. control group; **P<0.05, vs. smoking group.

### Quantitative real-time PCR

The gene expression of inflammatory markers, including interleukin-1β (IL-1β), interleukin-6 (IL-6), and tumor necrosis factor-α (TNF-α) were deteced by quantitative real-time PCR. Total RNA was extracted from left ventricular tissue using Trizol Reagent according to the manufacturer's instructions(Invitrogen, Karlsruhe, Germany). The concentration of RNA was quantified by measuring the absorbance at 260 nm. The reverse transcription mixture contained 1 μg of total RNA, 0.5 μg of oligo d(T) primer, 4 μl of 5×RT buffer, 0.5 mM deoxynucleotides, 50 U of RNase inhibitor and 200 U of reverse transcriptase (Promega, Madison, WI, USA) in a total volume of 20 μl. The reaction was carried out at 42°C for 1 h followed by heat inactivation at 95°C for 5 min. Real-time PCR was performed on the ABI 7300 Real Time PCR System following the manufacturer's instructions. The reaction mixture consisted of 12.5μl of SYBR Premix Ex Taq containing TaKaRa Ex Taq HS, dNTP mixture, Mg^2+^ and SYBR Green I (ABI, USA), 0.3 μM of primer, 1 μL template DNA and ddH_2_O filled up to 25 μL. The amplification reactions were carried out according to the following cycling protocol. Initial denaturation for 5 min at 95°C was followed by 40 cycles of 94°C for 30 s, 60°C for 30 s and 72°C for 40 s. The final elongation phase was performed for 10 min at 72°C. In this study, β-actin was used as internal control. The primer sequences used were as follows: IL-1β, forward primer 5′-TACCTATGTCTTGCCCGTGGAG-3′ and reverse primer 5′-ATCATCCCACGAGTCACAGAGG-3′; IL-6, forward primer 5′-GTCAACTCCATCTGCCCTTCAG-3′ and reverse primer 5′-GGCAGTGGCTGTCAACAACAT-3′; TNF-α, forward primer 5′-ACAAGGCTGCCCCGACTAT-3′ and reverse primer 5′-CTCCTGGTATGAAGTGGCAAATC-3′; β-actin, forward primer 5′-TGTCACCAACTGGGACGATA-3′ and reverse primer 5′-AACACAGCCTGGATGGCTAC-3′. The fold change of mRNA was analyzed using the 2^−ΔΔCt^ method [23].

### Detection of inflammatory cytokines by ELISA

The serum levels of inflammatory markers, including IL-1β, IL-6, and TNF-α, were detected using the commercial enzyme-linked immunosorbent assay (ELISA) kits (Bender Medsystems Diagnostics GmbH, Vienna, Austria). All experimental procedures were performed according to the manufacturer's instructions.

**Figure 4:**
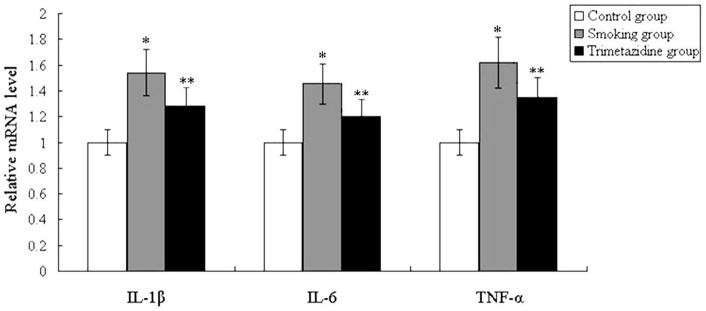
The gene expression of inflammatory markers were deteced by quantitative real-time PCR. The mRNA expression of IL-1β, IL-6, and TNF-α were up-regulated in the smoking group compared to the control group, while the expression of these markers were down-regulated in the trimetazidine group compared to the smoking group. *P<0.05, vs. control group; **P<0.05, vs. smoking group.

### Statistical analysis

Data were presented as mean ± SD and differences between groups were analyzed using one-way ANOVA with the SPSS 15.0 (SPSS, Inc., Chicago, IL, USA). Scheffé post hoc test was used if the ANOVA was significant. A P value <0.05 was considered statistically significant.

## Results

The echocardiographic data are presented in Table 1. Significant increases in LVEDD and LVESD were found in the smoking group compared to the control group. FS and EF in the smoking group were lower than in the control group, while these two variables in the trimetazidine group were higher than in the smoking group. In addition, there were no statistically significant differences in E/A and Ea/Aa ratios among these three groups.

**Table 4:** Serum levels of inflammatory cytokines.

	Control group	Smoking group	Trimetazidine group
IL-1β (pg/ml)	3.72±0.49	5.94±0.72*	4.58±0.65**
IL-6 (pg/ml)	26.48±5.32	42.53±8.76*	34.75±7.14**
TNF-α (ng/ml)	0.82±0.14	1.41±0.23*	1.13±0.19**

IL-1β: interleukin-1β; IL-6: interleukin-6; TNF-α: tumor necrosis factor-α;. Data are expressed as mean ± SD. *P<0.05, vs. control group; **P<0.05, vs. smoking group.

The morphometric data are presented in Table 2. LVMI, CSA and ICVF in the smoking group were significantly higher than in the control group, whereas these three variables were significantly lower in the trimetazidine group compared to the smoking group.

The HE-stained images of left ventricular tissue are shown in Fig. 1. Cardiomyocytes in the control group were orderly arranged, and the nuclei were lightly stained and located in the center of muscle fibers. Thickening and lengthening of myocardial fibers could be observed in the smoking group, wherein the nuclei were darkly stained, showing local tissue fibrosis and inflammatory cell infiltration. Cardiomyocyte hypertrophy, cellular degeneration and inflammatory cell infiltration were significantly improved in the trimetazidine group by contrast with those in the smoking group.

Oxidative stress was evaluated by detecting MDA, SOD and GSH-Px in the supernatant of left ventricular tissue and the results are presented in Table 3. Our findings showed a significant increase in MDA level and decreases in SOD and GSH-Px activities in the smoking group compared to the control group, while in the trimetazidine group, a significant decrease in MDA level and increases in SOD and GSH-Px activities were found compared to the smoking group.

Cardiomyocyte apoptotic rate was determined by flow cytometry with Annexin V/PI staining and the results are shown in Figs. 2 and 3. The apoptotic rate in the smoking group was significantly higher than in the control group, whereas apoptotic rate in the trimetazidine group was significantly lower than in the smoking group.

The gene expression of inflammatory markers were deteced by quantitative real-time PCR and the results are shown in Fig. 4. Our findings indicated that the mRNA expression of IL-1β, IL-6, and TNF-α were up-regulated in the smoking group compared to the control group, whereas the expression of these markers were down-regulated in the trimetazidine group compared to the smoking group.

The serum levels of inflammatory cytokines were detected by ELISA and the results are presented in Table 4. The concentrations of IL-1β, IL-6, and TNF-α in the smoking group were significantly higher than in the control group, whereas the levels of these markers in the trimetazidine group were significantly lower than in the smoking group.

## Discussion

In the present study, we demonstrated for the first time that trimetazidine has protective effects against smoking-induced left ventricular remodeling via attenuating oxidative stress, apoptosis, and inflammation.

To date, there have been few studies examining the adverse effects of smoking on left ventricular remodeling. Although smoking affects the entire cardiovascular system, it has been generally accepted that the most adverse effects were on the vasculature and included endothelial dysfunction and accelerated atherosclerosis [24]. However, the associations between smoking and reduced left ventricular function and greater left ventricular mass have been confirmed in more recent large population-based epidemiological studies using magnetic resonance imaging to evaluate myocardial structure and function [25], [26]. These data are further substantiated by our findings and those of others [7]–[9], which demonstrate that cigarette smoke exposure is associated with left ventricular remodeling.

In the present study, our results demonstrate that exposure to cigarette smoke could induce myocardial hypertrophy and ventricular enlargement which play a critical role in the remodeling process. One of the most striking characteristics of cardiac remodeling is a progressive decrease in ventricular function. Initially, due to cell growth, the remodeling process may help to maintain or restore ventricular function. Chronically, however, biochemical, genetic, and structural alterations occur, resulting in progressive ventricular dysfunction. In agreement with this concept, our study indicated that cigarette smoke exposure is associated with a significant decrease in left ventricular systolic function.

Trimetazidine, a piperazine derivative used as an anti-anginal agent, improves myocardial glucose utilization through inhibition of fatty acid metabolism. A previous study has reported that trimetazidine treatment was associated with a significant improvement of left ventricular function and the remodeling process in patients with ischaemic dilated cardiomyopathy [27]. In the present study, we established a smoking rat model to investigate the protective effects of trimetazidine against smoking-induced left ventricular remodeling and explore the potential mechanisms involved.

Oxidative stress, defined as an excess production of reactive oxygen species (ROS), has been shown to be involved in the pathophysiology of ventricular remodeling and associated with left ventricular dysfunction [28]–[30]. Excessive ROS generation triggers cell dysfunction, lipid peroxidation, and DNA mutagenesis and can lead to irreversible cell damage or death [31]–[33]. ROS can directly impair contractile function by modifying proteins central to excitation-contraction coupling [34]. Moreover, ROS activate a broad variety of hypertrophy signaling kinases and transcription factors [35]. They also stimulate cardiac fibroblast proliferation and activate the matrix metalloproteinases, leading to the extracellular matrix remodeling [36], [37]. In the present study, oxidative stress was evaluated by detecting MDA, SOD and GSH-Px in the supernatant of left ventricular tissue. Our findings suggested that trimetazidine could significantly decrease smoking-induced oxidative stress, which might be one of the protective mechanisms against smoking-induced left ventricular remodeling.

Apoptosis, a highly regulated biological process, plays a critical role in the pathogenesis of ventricular remodeling [38], [39]. A previous study has reported that cardiomyocyte apoptosis is related to left ventricular dysfunction and remodeling in dilated cardiomyopathy [40]. It has been revealed that myocardial apoptosis is strongly associated with unfavorable left ventricular remodeling and early symptomatic post-infarction heart failure [41]. In the present study, cardiomyocyte apoptotic rate was determined by flow cytometry with Annexin V/PI staining. Our results suggested that trimetazidine could significantly reduce smoking-induced apoptosis, which might be another protective mechanism against smoking-induced left ventricular remodeling.

Inflammation also plays an important role in the pathogenesis of ventricular remodeling. It has been demonstrated that inflammatory cytokines, including IL-1β, IL-6, and TNF-α, increase remarkably after myocardial infarction and are involved in the subsequent left ventricular remodeling [42]. A previous study has revealed that pathophysiologically relevant concentrations of TNF-α promote progressive left ventricular dysfunction and remodeling in rats [43]. In the present study, gene expression and serum levels of inflammatory markers, including IL-1β, IL-6, and TNF-α, were deteced to evaluate the extent of systemic inflammation. Our findings suggested that trimetazidine could significantly reduce smoking-induced inflammation, which might be an important protective mechanism against smoking-induced left ventricular remodeling.

In conclusion, our study demonstrates that trimetazidine protects against smoking-induced left ventricular remodeling via attenuating oxidative stress, apoptosis, and inflammation, which will provide valuable insights into the prevention and treatment of left ventricular remodeling induced by smoking.
